# Comparative habitat-associated patterns of pathomorphological changes in the internal organs of the European brown hare (*Lepus europaeus Pall*.)

**DOI:** 10.14202/vetworld.2026.1387-1401

**Published:** 2026-04-12

**Authors:** Nikola Mihajlović, Darko Marinković, Dejan Beuković, Vukan Lavadinović, Stefan Stepić, Blagoje Stojković, Zoran Popović

**Affiliations:** 1 Department of Animal Science, Faculty of Agriculture, University of Belgrade, Belgrade, Serbia; 2 Department of Pathology, Faculty of Veterinary Medicine, University of Belgrade, Belgrade, Serbia; 3 Department of Animal Science, Faculty of Agriculture, University of Novi Sad, Novi Sad, Serbia; 4 Chair of Forest Resource Utilization, Faculty of Forestry, University of Belgrade, Belgrade, Serbia

**Keywords:** agricultural habitat, bioindicator species, European brown hare, habitat type, histopathology, organ lesions, pathomorphological changes, wildlife health monitoring

## Abstract

**Background and Aim:**

The European brown hare (*Lepus europaeus* Pall.) is an ecologically significant game species widely used as a bioindicator of environmental conditions because of its sensitivity to habitat changes and human activities. Pathomorphological changes in internal organs offer valuable insights into the health of wildlife populations and may indicate exposure to various environmental stressors. However, research that evaluates multiple organ systems simultaneously in relation to habitat type within the same spatiotemporal context remains limited. Therefore, this study aimed to compare the frequency and distribution of pathomorphological changes in the liver, lungs, spleen, and kidneys of European brown hares from agricultural and periurban or industrial hunting areas in central Serbia during the same hunting season. The study also sought to identify potential habitat-related patterns in organ alterations.

**Materials and Methods:**

The study was carried out during the 2017/2018 hunting season on 74 European brown hares collected from four hunting grounds in central Serbia, classified as agricultural or periurban/industrial based on land use. Macroscopic and histopathological examinations included the liver (n = 74), lungs (n = 73), spleen (n = 61), and kidneys (n = 65). Tissue samples were fixed in 10% neutral buffered formalin, processed with routine paraffin techniques, sectioned at 3–5 µm, and stained with hematoxylin and eosin. The frequency of pathological changes was analyzed using Pearson’s chi-square test and Fisher’s exact test, with statistical significance set at p < 0.05. Odds ratios with 95% confidence intervals were calculated to evaluate the association between lesion presence and hunting ground type.

**Results:**

Significant habitat-related differences were observed in several organs. Dystrophic liver changes were more common in hares from agricultural hunting grounds (OR = 0.33; p = 0.026). In the lungs, bronchiolitis (OR = 0.32; p = 0.025) and emphysema (OR = 0.10; p = 0.010) occurred more frequently in hares from periurban or industrial areas. In the spleen, lymphoid hyperplasia (OR = 0.22; p = 0.008) and splenomegaly (OR = 0.06; p = 0.0001) were significantly more prevalent in agricultural habitats. No statistically significant link between hunting ground type and kidney lesions was found.

**Conclusion:**

Distinct habitat-related patterns of pathomorphological changes were observed in the European brown hare, with liver and spleen abnormalities mainly in agricultural areas and lung lesions in periurban or industrial regions. These findings suggest that organ-based pathology can be a useful additional tool for wildlife health monitoring and may offer early signs of adverse environmental conditions without establishing direct cause-and-effect relationships.

## INTRODUCTION

As a widely distributed and abundant species, the European brown hare (*Lepus europaeus* Pall.) is native to most parts of Europe and Western Asia and has been introduced to other continents [1, 2]. Due to its ecological and economic importance, this species is one of the key game animals in hunting management [3, 4]. As a typical inhabitant of open landscapes, the European brown hare is highly sensitive to changes in land-use structure and intensity, and its population density and spatial distribution are closely linked to agroecosystem characteristics and the level of agricultural intensification [5–8]. The species is also of particular interest in wildlife health research because examining organs and tissues provides assessment of exposure to various external factors and their potential effects on the organism [9–12]. For these reasons, the European brown hare is considered a species of conservation concern, especially in Europe [13].

However, a decline in the population of the European brown hare has been observed since the 1960s in several European countries [5, 14–17], and recent studies confirm that this negative trend continues across Europe [18, 19]. The decrease in hare numbers results from multiple interconnected factors, most commonly including changes in agricultural practices, climate conditions, and predation pressure [20, 21]. The development of intensive agriculture, characterized by increased mechanization, landscape homogenization, extensive use of agrochemicals, and the expansion of monoculture cropping systems, has led to habitat degradation and a reduction in natural food resources and shelter for the European brown hare [5–8, 14, 22]. Additionally, urbanization and infrastructure development contribute to habitat fragmentation and the loss of diverse environments, which in some cases causes local extinctions of populations [17]. Under such conditions, the European brown hare faces increased ecological stress, while the buildup of toxic substances in organs, especially heavy metals, can cause tissue damage and impair health, thereby negatively impacting individual survival and overall population numbers [23, 24].

Furthermore, many authors have stressed that diseases play a significant role in the decline of European brown hare populations, with European brown hare syndrome being identified as the most impactful disease affecting this species [25–27]. According to Vizzarri *et al*. [28], European brown hare populations are currently at record low levels. Pathomorphological changes in the organs of wild animals serve as a reliable indicator of exposure to various harmful environmental factors [29, 30]. Therefore, ongoing monitoring of health status and the presence of organ lesions in hare populations is essential to identify factors responsible for their development [11, 31]. Such findings can provide a crucial basis for planning management strategies and activities aimed at reducing negative trends in European brown hare populations. In this context, analyzing pathomorphological changes in hare organs may help improve understanding of how different habitat types and levels of human activities affect the health and vitality of this species’ populations [9, 11, 12]. Besides its importance for assessing population health, previous studies suggest that the European brown hare can act as a useful bioindicator, or biomonitor, of environmental conditions, since changes in its tissues and organs can reflect the level of environmental stress in its habitats [9, 11, 12, 32, 33].

Although many studies have examined the population dynamics, ecological needs, and disease prevalence in *L. europaeus* Pall., most research has concentrated on either specific environmental factors, individual organ systems, or populations that are geographically and temporally variable. Past research often assessed pathological findings without directly comparing different habitat types within the same region and time frame, which limits the ability to distinguish habitat-related patterns from seasonal or spatial differences. Additionally, many studies have relied solely on field observations or toxicological tests, while comprehensive pathomorphological assessments involving multiple internal organs are still relatively rare. The absence of studies that combine histopathological data from various organ systems under similar ecological conditions makes it challenging to determine whether certain organ alteration patterns are related to land-use differences and human impact. Moreover, few investigations have compared populations from agricultural areas and periurban or industrial environments within the same hunting season, which is essential to reduce the effects of climate and timing variables. Consequently, there is a need for a systematic, multi-organ, habitat-focused approach that enables the evaluation of potential differences in the frequency and distribution of pathomorphological changes in the European brown hare under controlled spatiotemporal conditions.

In this context, the aim of this study was to compare the frequency and distribution of pathomorphological changes in the liver, lungs, spleen, and kidneys of *L. europaeus* Pall. from hunting grounds characterized by different dominant habitat types, classified as agricultural and periurban/industrial, within the same geographic region of central Serbia and during the same hunting season. An additional goal was to determine whether distinct habitat-related patterns of organ alterations could be identified and whether certain organ systems are more sensitive to environmental conditions linked to land-use structure and human activity. Using a cross-sectional comparative design based on histopathological examination of multiple organs, this study aimed to enhance the internal comparability of results and reduce the influence of seasonal, climatic, and regional variability. The findings are expected to improve understanding of the relationship between habitat features and the health of European brown hare populations and to assess the potential of pathomorphological analysis as a supplementary tool for wildlife health monitoring and environmental assessment, without implying direct cause-and-effect relationships with specific environmental factors.

## MATERIALS AND METHODS

### Ethical approval

This study did not need ethical approval because it was based solely on post-mortem organ samples collected from *L. europaeus* Pall. legally hunted during the regular season, in full compliance with the Wildlife and Hunting Law of the Republic of Serbia (“Official Gazette of RS”, Nos. 18/10, 95/18, and 92/23) [34]. No live animals were used, and none were killed specifically for research. The animals were harvested independently of the study, following standard hunting practices, and the researchers had no influence on the selection or culling of animals. All procedures were carried out according to applicable national laws on wildlife management and animal protection.

### Study period and location

The study was conducted during the 2017/2018 hunting season, from October to December, in accordance with the applicable regulations of the Republic of Serbia. Samples were collected as part of routine hunting activities, ensuring a uniform sampling period without seasonal variation within the year.

**Figure 1:**
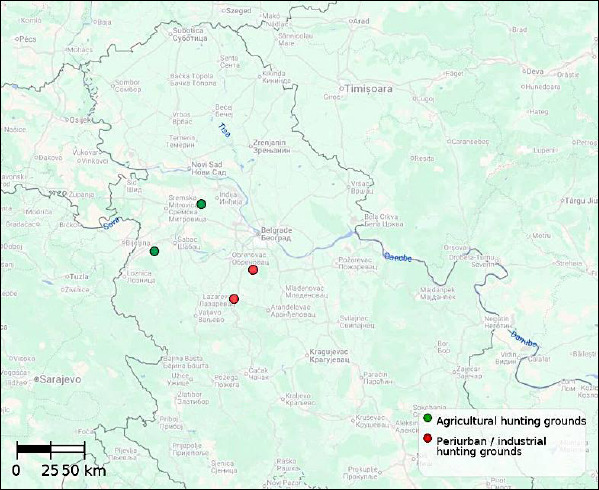
Map of Central Serbia illustrating the locations of the investigated hunting grounds.

The investigation included hunting grounds located in central Serbia, specifically Barajevo, Lazarevac, Žarkovci, and Lipolist (Figure 1). These hunting grounds were selected because they are situated within the same geographic region and climatic zone, yet differ in land-use structure and the degree of anthropogenic influence. Such site selection enabled a comparison of pathomorphological findings in *L. europaeus* across different environmental types within the same temporal framework.

### Study design

The study was designed as a cross-sectional observational study with a comparative approach. The aim was to determine whether the frequency and patterns of pathomorphological changes in the organs of the European brown hare (*Lepus europaeus* Pall.) vary between individuals from hunting grounds characterized by different dominant environmental types, defined as agricultural and periurban/industrial. Accordingly, samples were collected from multiple hunting grounds within the same geographic area, which were pre-classified into two categories based on the dominant land use. The study was carried out during a single hunting season as part of routine hunting activities, without any experimental manipulation.

### Study population and animal characteristics

All *L. europaeus* Pall. organs from all harvested individuals collected in the examined hunting grounds during the 2017/2018 hunting season were included in the study, totaling 74 animals. Of these, 25 came from agricultural hunting grounds, while 49 were from periurban or industrial hunting grounds. The sample consisted of all available individuals harvested during that hunting season, without any additional selection.

Differences in sample size among hunting ground types resulted from several interconnected factors, including variations in population status, abundance, and culling plans across individual hunting grounds. These factors also affected the number of organized hunting days during the season and harvest success, which was further influenced by terrain features and the number of participants in hunting activities. Therefore, the final sample size and its distribution by hunting ground type reflect the actual management practices and field conditions during the study period.

Following harvest, in most cases only individual organs and the head were submitted, which allowed for age determination, while sex could be identified only when reproductive organs were available (Table 1). Age was estimated by measuring the mass of the eye lenses using a modified method described by Mihajlović et al. [4], where individuals were classified as young (≤1 year) or adult (>1 year), with a lens mass threshold of 280 mg, in accordance with the referenced methodology. Sex was determined based on a macroscopic examination of the submitted gonads when available. Data on body condition or carcass mass were not collected, as complete carcasses were unavailable in most cases.

**Table 1:** Sex and age distribution of European brown hares (*Lepus europaeus*) sampled from agricultural and periurban/ industrial hunting grounds in Central Serbia.

Hunting ground type	n	Male	Female	Unknown	Juvenile	Adult	Unknown
Agricultural	25	6	7	12	7	6	12
Periurban/industrial	49	15	16	18	16	16	17
Total	74	21	23	30	23	22	29

The main goal of the study was to compare the frequency and distribution of pathomorphological changes across different hunting ground types. The evaluation of how individual biological factors like sex and age affected these changes was not a primary focus. Because of limited data availability and small sample sizes in some categories, sex and age were not incorporated into multifactorial statistical models. Instead, they were recorded descriptively and considered potential confounding factors when interpreting the results.

### Histopathological examination

In most cases, individual organs from harvested *L. europaeus* were submitted for histopathological analysis, while complete carcasses were available in only a few cases. Organs or tissue samples were transported under refrigerated conditions (4°C–8°C) and delivered to the laboratory within 24 h of harvesting. Depending on the sample arrival times, laboratory processing and preparation of histological sections began immediately upon receipt of the material to preserve tissue structure and reduce the risk of postmortem degradation and autolysis.

Because not all organs were available in every case, and due to mechanical damage during harvesting or partial degradation of some samples, the number of histopathologically examined samples varied by organ. In total, samples from 74 livers, 73 lungs, 61 spleens, and 65 kidneys were collected and analyzed.

Upon receipt, a routine macroscopic examination of the organs was performed to assess their overall appearance and guide further histopathological analysis. Since many pathological changes in the examined organs cannot be reliably identified visually, the systematic analysis in this study was mainly based on histopathological examination. Macroscopic changes were recorded when clearly visible but were not evaluated using standardized or quantitative methods, except for splenomegaly, which was documented as a binary macroscopic finding (presence or absence) and included in descriptive and statistical analyses. Conversely, for the liver, lungs, and kidneys, all pathological changes analyzed were confirmed through histopathology, and macroscopic findings in these organs were not considered as separate variables.

Liver, lung, spleen, and kidney samples intended for histopathological analysis were fixed in 10% neutral buffered formalin for 72 h, using a fixative-to-tissue ratio of at least 10:1. After fixation, samples were processed with an automatic tissue processor KD-TS3D (Kedee, PR China), which included dehydration through increasing ethanol concentrations (70%, 96%, and 100%), clearing in xylene, and paraffin embedding. The processed samples were then embedded in paraffin blocks.

Paraffin blocks were sectioned at 3–5 μm thickness using a rotary microtome Leica RM 2235 (Leica Biosystems, USA). Sections were mounted on glass slides and dried at 56°C for 24 h in a Binder E/B28 thermostat (Binder, Germany). After deparaffinization in xylene and rehydration through decreasing alcohol concentrations (100%, 96%, and 70%), sections were stained with hematoxylin and eosin (H&E). Hematoxylin was applied for 5 minutes, followed by differentiation in acid alcohol, then counterstained with eosin for 3 minutes, with water rinses between steps. After final dehydration through increasing alcohol concentrations and treatment with xylene, the slides were mounted and coverslipped. Histological sections were examined under a BX51 light microscope (Olympus Optical, Japan) and photographed using an Olympus Color View III® digital camera (Olympus, Japan). All histological procedures followed standard, widely accepted techniques for paraffin-embedded sections, as described in typical histological methodological literature [35].

Histopathological evaluation was conducted using a blinded assessment method. All slides were examined by a single trained veterinary pathologist who was unaware of the samples’ source regarding hunting ground type, thus minimizing the risk of classification bias.

Parasitic lesions were identified based on characteristic morphological features observed during histopathological examination. No additional parasitological, coprological, or molecular confirmation methods were performed; therefore, the identification of parasitic species (e.g., *Dicrocoelium dendriticum* and *Taenia pisiformis*) was considered presumptive and relied solely on morphological criteria.

During histopathological evaluation, all observed changes in the examined organs were systematically recorded. The presence or absence of specific histopathological lesions was documented for each analyzed organ sample, and the data were compiled into a database for further analysis of lesion frequency and distribution by organ.

### Statistical analysis

Statistical analysis was conducted to compare the frequency of pathomorphological changes between agricultural and periurban/industrial hunting grounds. Analyses were performed separately for each organ and lesion type, based on predefined, biologically relevant comparisons rather than an exploratory approach involving testing many variables. The choice of statistical tests was driven by the categorical nature of the data, as each pathomorphological change was considered a binary outcome (presence/absence), making tests of independence in 2×2 contingency tables the most suitable method for this analysis.

For each lesion included in the inferential analysis, a 2 × 2 contingency table (hunting ground type × lesion presence) was created. Pearson’s chi-square test was used when all expected frequencies in the table were ≥5, whereas Fisher’s exact test was employed when at least one expected frequency was <5 [36, 37]. The threshold for statistical significance was set at p < 0.05. Although multiple statistical tests were conducted, no formal correction for multiple comparisons was applied, since all analyzed pathomorphological changes were predetermined based on pathological and biological relevance and were considered within clearly defined, organ-specific hypotheses. Using conservative corrections in this study design could increase the risk of type II errors and lead to underestimating biologically significant differences between the groups.

Pathomorphological changes that had a low overall frequency were not included in inferential testing but were instead described. This method was chosen because of limited statistical power to detect differences in rare lesions and the risk of unstable estimates in contingency tables with small sample sizes, which could lead to misleading conclusions.

To improve the understanding of findings beyond just statistical significance, odds ratios (ORs) with their 95% confidence intervals (CI) were calculated for all changes included in the inferential analysis.

Statistical analyses were conducted with IBM SPSS Statistics software, version 26.0 (IBM Corp., Armonk, NY, USA).

## RESULTS

### Liver

Among the examined pathomorphological changes in the liver, an association was observed between dystrophic changes and hunting ground type, with hares from periurban and industrial hunting grounds showing significantly lower odds of developing these changes compared to those from agricultural hunting grounds (OR = 0.33; 95% CI: 0.12–0.89; p = 0.026). No link with hunting ground type was found for other liver changes, including hyperemia, hepatitis, and distomatosis/cholangitis, and the estimated odds ratios were accompanied by wide confidence intervals that included the value of 1.

**Figure 2:**
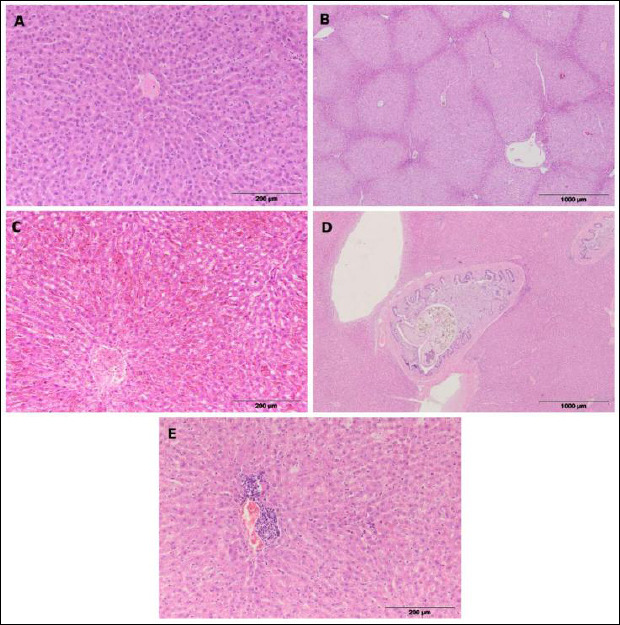
Representative histological sections of the liver in European brown hares. (a) Normal liver parenchyma with no visible lesions; (b) hepatic dystrophy or degeneration; (c) hepatic hyperemia or congestion; (d) hepatic distomatosis with cholangitis or pericholangitis (liver fluke infestation); (e) lymphohistiocytic hepatitis.

Macroscopically, the livers were slightly enlarged, lighter in color, and fragile in texture. The main histopathological changes were dystrophic, meaning degenerative and necrotic, including intracellular edema marked by cytoplasmic clouding and vacuolization of hepatocytes, as well as necrobiotic alterations in hepatocyte nuclei such as karyopyknosis, karyorrhexis, and karyolysis, which are typical of necrosis (Figure 2b).

Along with degenerative changes, hyperemia was observed in the liver. Macroscopically, it appeared as a darker coloration of the organ and a greater amount of blood released from the cut surface. Histopathologically, it was characterized by engorgement of blood vessels (veins and arteries) and significant congestion of sinusoidal capillaries (Figure 2c).

In several individuals, inflammation of the hepatic parenchyma, known as hepatitis (Figure 2e), was observed, characterized by infiltration of mononuclear cells (lymphocytes, macrophages, and plasma cells) within the liver tissue.

Changes were also seen in the bile ducts, visible externally as wall thickening and microscopically by the presence of adult small liver fluke (*Dicrocoelium dendriticum*) within the bile duct lumen. This was accompanied by inflammation of the bile ducts (cholangitis) and the surrounding tissue (pericholangitis) of parasitic origin (Figure 2d). Parasitic cholangitis ranged from proliferative, showing epithelial growth of the bile ducts, to destructive, involving destruction and shedding of the bile duct epithelium with cellular debris in the lumen. Pericholangitis was marked by infiltration of mononuclear cells (lymphocytes, macrophages, plasma cells), eosinophilic granulocytes, and multinucleated giant cells. These findings confirm hepatic distomatosis (liver fluke infestation).

In addition to the aforementioned liver lesions, larval forms of the carnivore tapeworm (*Taenia pisiformis*) were macroscopically observed on the liver and omentum, appearing as cystic formations (*Cysticercus pisiformis*).

### Lungs

Analysis of pathomorphological changes in the lungs showed differences in the frequency of bronchiolitis and emphysema based on hunting ground type. These lesions were more commonly seen in hares from periurban or industrial hunting grounds, while hares from agricultural hunting grounds had lower odds of developing them, as indicated by the estimated odds ratios (bronchiolitis: OR = 0.32; 95% CI: 0.11–0.88; emphysema: OR = 0.10; 95% CI: 0.01–0.80). No link was found between hunting ground type and other tested pulmonary changes, including interstitial pneumonia, hyperemia, and hemorrhages, and the estimated odds ratios had confidence intervals that included the value of 1 (Table 3).

**Table 2:** Prevalence of pathomorphological lesions in the liver of European brown hares from agricultural (n = 25) and periurban/industrial (n = 49) hunting grounds, including odds ratios (OR), 95% confidence intervals (CI), and p-values.

Lesion	Agricultural (n)	%	Periurban/ industrial (n)	%	Total (%)	OR (95% CI)	p-value
Dystrophy	16	64.0	18	36.7	45.9	0.33 (0.12–0.89)	0.026
Hyperemia	4	16.0	14	28.6	24.3	2.10 (0.61–7.23)	0.233
Distomatosis / cholangitis–pericholangitis	2	8.0	10	20.4	16.2	2.95 (0.59–14.65)	0.204
Hepatitis	4	16.0	6	12.2	13.5	0.73 (0.19–2.88)	0.725
Granulomas	1	4.0	4	8.2	6.8	—	—
Autolysis	0	0.0	2	4.1	2.7	—	—
Cysticercosis	0	0.0	1	2.0	1.4	—	—

**Table 3:** Prevalence of pathomorphological lesions in the lungs of European brown hares from agricultural (n = 24) and periurban/industrial (n = 49) hunting grounds, including odds ratios (OR), 95% confidence intervals (CI), and p-values.

Lesion	Agricultural (n)	%	Periurban/industrial (n)	%	Total (%)	OR (95% CI)	p-value
Bronchiolitis	8	33.3	30	61.2	52.1	0.32 (0.11–0.88)	0.025
Interstitial pneumonia	13	54.2	16	32.7	39.7	2.44 (0.90–6.63)	0.078
Hyperemia	4	16.7	15	30.6	26.0	0.45 (0.13–1.56)	0.202
Emphysema	1	4.2	15	30.6	21.9	0.10 (0.01–0.80)	0.010
Hemorrhages	4	16.7	12	24.5	21.9	0.62 (0.18–2.16)	0.448
Peribronchiolitis/peribronchitis	0	0.0	3	6.1	4.1	—	—
Granulomas	0	0.0	2	4.1	2.7	—	—

— indicates that statistical testing was not performed due to the low frequency of the lesion.

**Figure 3:**
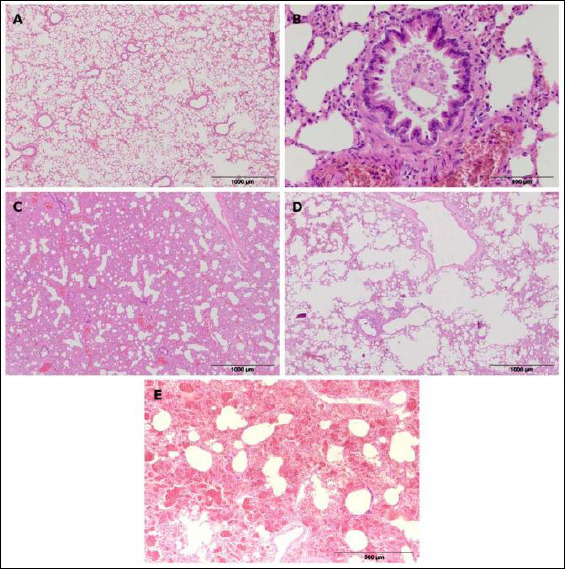
Representative histological sections of the lungs in European brown hares. (a) Normal pulmonary tissue; (b) necrotizing obstructive bronchiolitis; (c) interstitial pneumonia; (d) pulmonary emphysema; (e) pulmonary hemorrhage.

Bronchiolitis was the most common pathological lesion, mainly appearing as necrotizing obstructive bronchiolitis (Figure 3b), characterized by the filling of the bronchiolar lumen with desquamated necrotic epithelial cells, known as “ghost cells,” and cellular debris. In some individuals, a proliferative form of bronchiolitis was observed, marked by papillary proliferation of the bronchiolar epithelium. Peribronchiolitis and peribronchitis represented localized inflammatory processes surrounding the bronchioles and bronchi, characterized by infiltration of lymphocytes, macrophages, and plasma cells.

Interstitial pneumonia (Figure 3c) was a common finding and was characterized by thickening of the alveolar walls caused by the proliferation of type II pneumocytes, narrowing of the alveolar spaces, and increased cellularity of the interstitium, resulting in a typical “Swiss cheese” appearance of the lungs.

Among circulatory disturbances, hyperemia was the most common finding, characterized by engorgement of larger blood vessels and capillaries within the alveolar walls. Hemorrhages (Figure 3e) were evident as blood within the alveoli, bronchioles, and pulmonary interstitium.

In a smaller number of cases, changes in the form of emphysema (Figure 3d) were observed, characterized by increased airiness of the pulmonary parenchyma, with enlarged alveoli and thinning of the alveolar septa.

In some cases, granulomas were observed in the pulmonary parenchyma, composed of macrophages, epithelioid and multinucleated giant cells, lymphocytes, and a connective tissue capsule. In several individuals, cross-sections of parasites or eggs with a thick cuticle were detected in the central part of the granulomas, indicating parasitic granulomas.

### Spleen

Variations in the frequency of lymphoid hyperplasia and splenomegaly in the spleen were noted depending on the type of hunting ground. These conditions were more commonly observed in hares from agricultural hunting areas, while hares from periurban or industrial hunting zones had significantly lower odds of developing them, as shown by the estimated odds ratios (hyperplasia: OR = 0.22; 95% CI: 0.07–0.70; splenomegaly: OR = 0.06; 95% CI: 0.01–0.32). Other pathological changes in the spleen did not show any association with the hunting ground type and are described narratively (Table 4).

**Table 4:** Prevalence of pathomorphological lesions in the spleen of European brown hares from agricultural (n = 25) and periurban/industrial (n = 36) hunting grounds, including odds ratios (OR), 95% confidence intervals (CI), and p-values.

Lesion	Agricultural (n)	%	Periurban/industrial (n)	%	Total (%)	OR (95% CI)	p-value
Hyperplasia	12	48.0	6	16.7	29.5	0.22 (0.07–0.70)	0.008
Splenomegaly	12	48.0	2	5.6	23.0	0.06 (0.01–0.32)	0.0001
Hyperemia	3	12.0	3	8.3	9.8	—	—
Leukosis	1	4.0	2	5.6	4.9	—	—
Hemosiderosis	0	0.0	2	5.6	3.3	—	—

— indicates that statistical testing was not performed due to the low frequency of the lesion.

**Figure 4:**
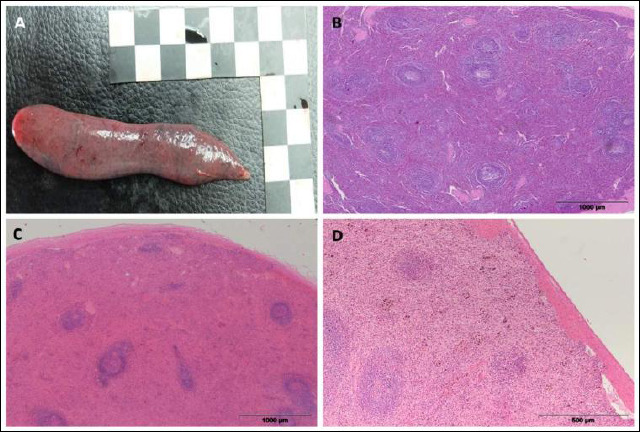
Representative histological sections of the spleen in European brown hares showing pathological changes. (a) Splenomegaly; (b) lymphoid hyperplasia; (c) splenic hyperemia/congestion; (d) hemosiderosis.

Splenomegaly (Figure 4a) was a common macroscopic finding, characterized by an enlarged organ and changed tissue consistency. The spleen’s surface was smooth, while the cut surface showed dark red, moist tissue. In most cases, splenomegaly was linked to hyperplastic changes in lymphoid tissue, which histologically included lymphocyte proliferation within the white pulp, enlarged lymphoid follicles, and increased mitotic activity.

In some individuals, hyperplasia (Figure 4b) was accompanied by a loss of clear structural organization of the spleen, with indistinct borders between the white and red pulp, indicating significant immunoreactivity of the lymphoid tissue.

Among circulatory changes, hyperemia (Figure 4c) was observed, characterized by engorgement of blood vessels and sinusoids within the red pulp. In smaller areas of the samples, accumulation of brown pigment within macrophages was also noted, indicating splenic hemosiderosis (Figure 4d).

In rare cases, a proliferative process of lymphoid tissue with marked mitotic activity and loss of the normal cytological and histological architecture of the organ was observed, indicating a neoplastic lesion, i.e., leukosis.

### Kidneys

Unlike the liver, lungs, and spleen, no clear link was found between the examined kidney changes and hunting ground type. Although variations in the frequency of tubulonephrosis, nephritis, and hyperemia were noted among the groups, the estimated odds ratios had wide confidence intervals that included the value of 1, suggesting no reliable association with hunting ground type (Table 5).

**Table 5:** Prevalence of pathomorphological lesions in the kidneys of European brown hares from agricultural (n = 23) and periurban/industrial (n = 42) hunting grounds, including odds ratios (OR), 95% confidence intervals (CI), and p-values.

Lesion	Agricultural (n)	%	Periurban/industrial (n)	%	Total (%)	OR (95% CI)	p-value
Tubulonephrosis	18	78.3	28	66.7	70.8	0.56 (0.16–1.96)	0.326
Nephritis (interstitial / glomerular)	4	17.4	9	21.4	20.0	1.30 (0.36–4.73)	0.758
Hyperemia	3	13.0	9	21.4	18.5	1.82 (0.44–7.47)	0.515
Hemorrhages	0	0.0	1	2.4	1.5	—	—

— indicates that statistical testing was not performed due to the low frequency of the lesion.

**Figure 5:**
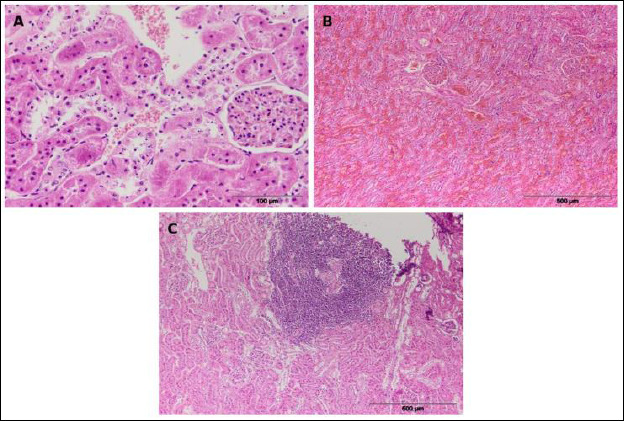
Representative histological sections of the kidneys in European brown hares showing pathological changes. (a) Tubulonephrosis; (b) renal hyperemia/ congestion; (c) interstitial nephritis.

Macroscopic and histopathological examination of the kidneys revealed that degenerative changes (tubulonephrosis) were the most common lesions observed (Figure 5a). The alterations affected the epithelium of the renal tubules, with microscopic evidence of vacuolar degeneration and cytoplasmic clouding. In some cases, epithelial damage was more severe, accompanied by tubulonecrosis with necrobiotic changes in the nuclei, such as karyopyknosis, karyorrhexis, and karyolysis.

Among inflammatory lesions, nephritis was the most frequently observed, most often as interstitial nephritis (Figure 5c), which was histologically characterized by focal infiltrates of lymphocytes, plasma cells, and histiocytes within the interstitium. Changes in the glomeruli appeared as proliferative or exudative glomerulonephritis.

Circulatory disturbances included hyperemia (Figure 5b), characterized by blood vessel and capillary engorgement within the renal parenchyma, while hemorrhages were rare and limited to small areas of the cortex.

## DISCUSSION

### Pathomorphological patterns in relation to habitat type in the European brown hare

This study examined differences in the frequency and distribution of pathomorphological changes in *L. europaeus* Pall. based on hunting ground type. Since specific environmental exposure factors were not directly measured, the observed differences are viewed as patterns that occur more or less often within certain habitat types. This method allows for a comparison of population health profiles across various habitat categories without assuming direct cause–effect links with individual human-related factors. In this context, the findings can be seen within an ecopathological and One Health framework, where pathomorphological observations are analyzed in relation to spatial habitat features and land-use patterns as part of a larger approach to monitoring wildlife and environmental health.

### Pathomorphological pattern of the European brown hare in agricultural hunting grounds

In agricultural hunting grounds, this study identified a pathomorphological pattern mainly affecting the liver and spleen. Individuals from this type of hunting area more often showed dystrophic changes in the liver, as well as reactive changes in the spleen, notably splenomegaly and lymphoid hyperplasia, compared to hares from periurban and industrial hunting grounds. The liver and spleen are interconnected organs with essential roles in metabolism, detoxification, hematopoiesis, and immune defense; therefore, changes in these organs are often considered when assessing the overall health of animal populations.

Similar types of degenerative liver changes have been observed in animal studies examining the effects of chemical substances used in agriculture, including various pesticides, where disruptions in hepatocyte structure and metabolic alterations have been reported [38–40]. Additionally, field studies on *L. europaeus* from agricultural regions have documented liver findings that are discussed within the context of environmental conditions [11]. Such literature data offer a broader ecological perspective on the types of changes that may be seen in agroecosystems, without suggesting a direct cause-and-effect relationship in this study.

Reactive splenic changes, including splenomegaly and hyperplasia, are generally described in the literature as adaptive responses of the organism to nonspecific stressors, without a clear etiological link to a single environmental factor [30]. However, their increased occurrence within certain habitat types may reflect differences in the overall ecological burden faced by populations, which was observed in hares from agricultural hunting grounds in this study.

Besides the pattern described, other examined histological changes in the liver and spleen did not show clear or consistent differences related to hunting ground type. Their frequency varied between agricultural and periurban/industrial habitats without forming a recognizable habitat-specific pattern. In the literature, infla-mmatory liver lesions in *L. europaeus* are most often described as mild and nonspecific and are seen as secondary reactions to various infectious, nutritional, or environmental influences [41].

In this context, although the liver and spleen are organs where reactive and adaptive changes can appear under various stressors, the pattern observed in this study suggests that certain combinations of findings may be more commonly linked to specific hunting ground types. These habitat-related “micro-patterns” should not be seen as proof of a specific cause but rather as markers indicating differences in overall ecological stress between habitat types. These findings require further validation through larger studies and by combining pathomor-phological data with information on habitat-related factors, such as pesticides typical of agricultural hunting grounds and other pollutants that might be present in different environments.

### Pathomorphological pattern of the European brown hare in periurban/industrial hunting grounds

In periurban and industrial hunting grounds, the current study identified a pathomorphological pattern marked by more significant alterations in the respiratory system compared to agricultural hunting grounds. The lungs were the most affected organs, with bronchiolitis being the most prominent finding and the only lesion that clearly differed based on habitat type. Additionally, most other pulmonary lesions were generally more common in individuals from periurban and industrial hunting grounds, although without clear statistical significance, indicating a consistent but variable pattern of changes.

In this context, a study showing a higher occurrence of inflammatory lung changes in *L. europaeus* populations living near industrial pollution sources, such as proximity to thermal power plants, is particularly relevant [24]. Although the specific industrial sources and pollutant levels were not examined in this study, the higher rate of pulmonary lesions in periurban and industrial hunting areas suggests a similar habitat environment where the respiratory system may be a sensitive indicator of the population’s health.

Findings from other studies further support the idea that the respiratory system is often affected by disease changes in urban and industrial environments. In grey squirrels (*Sciurus carolinensis*) living in cities, focal inflammation, macrophage buildup, and lipoprotein pneumonia have been noted [42], while anthracosis and chronic inflammatory changes of the respiratory tract have been observed in urban dogs and other animals from human-impacted environments [43]. Experimental research in mice has shown that exposure to PM_2-5_ particles and urban aerosols can cause infiltration of inflammatory cells, damage to the bronchiolar lining, and thickening of alveolar walls [44, 45].

### Kidneys as an organ without a habitat-specific pathomorphological pattern

Unlike certain organ systems where habitat-related patterns of changes were observed in this study, the pathomorphological lesions of the kidneys in *L. europaeus* did not differ based on hunting ground type. The changes were seen in individuals from both habitat types in nearly the same proportions, with no clear pattern linking them to specific environmental conditions.

Similar findings have been observed in other *L. europaeus* populations, where renal changes are mostly seen as a nonspecific response to chronic systemic stress rather than as a result of specific habitat-related factors [27, 41]. Experimental studies in rodents have demonstrated that various types of chemical exposure, including pesticides and industrial pollutants, can cause similar degenerative and inflammatory renal changes [46–50], further supporting the idea that such a pathomorphological pattern is not unique to a particular environment.

In this context, the even distribution of renal lesions across different hunting ground types indicates that, in this study, the kidneys of *L. europaeus* do not serve as a reliable marker of habitat-specific differences, but instead reflect general systemic processes that may function under various ecological conditions.

### Practical implications for hunting management and wildlife health monitoring

Based on the observed habitat-specific pathomorphological patterns, this study’s results suggest that analyzing internal organs from harvested European brown hares can be a valuable additional tool for routine population health monitoring. Along with standard indicators used in hunting management, such as population size and age structure, pathomorphological findings can offer extra insights into the health challenges faced by populations living in different habitat types.

In agricultural hunting grounds, the observed patterns of liver and spleen changes suggest that these organs may be particularly useful for assessing the health profile of populations in agroecosystems, while in periurban and industrial hunting grounds, the respiratory system may serve as a more sensitive indicator of habitat conditions. This approach allows for monitoring activities to be tailored to the dominant habitat type while still providing a comprehensive evaluation of multiple organ systems.

From a hunting management perspective, such information can help make timely adjustments to management strategies, including regulating harvest pressure and improving habitat conditions, based on the population’s health status.

Although pathomorphological findings alone do not enable the identification of specific causal factors, their systematic monitoring can serve as an early warning of potentially adverse habitat conditions, before observable declines in population size or disruptions in population structure occur. In this context, the pathomorphological findings obtained in the present study may be regarded as an additional source of information for *L. europaeus* health monitoring, with potential value for early detection of harmful ecological trends.

Given that the European brown hare is a species sensitive to land-use changes and environmental stress, the identified habitat-specific patterns of internal organ changes may help in the early detection of chronic, low-level stressors in the environment. Although this approach does not provide direct evidence of environmental degradation, it can be valuable in guiding further focused research and monitoring efforts, as well as in enhancing the long-term management and conservation of *L. europaeus* populations.

### Limitations and future perspectives

This study has certain methodological limitations that should be considered when interpreting the results. First, the assessment of environmental exposure relied on classifying hunting grounds based on land-use type, without direct measurements of potential contaminants such as pesticide residues, heavy metals, or air pollutants. As a result, it is not possible to establish direct cause-and-effect relationships between specific human activities and observed histopathological changes; instead, the results can only be interpreted within the context of habitat-related patterns.

Another limitation is the lack of screening for infectious agents. Viral, bacterial, and parasitic infections, known to cause various pathological changes in *L. europaeus*, could not be ruled out with the methods used. As a result, potential infectious causes of some lesions are a confounding factor to consider when interpreting the findings. Parasitic lesions in this study were identified only based on morphological criteria, without additional parasitological, coprological, or molecular confirmation, so the identification of parasitic species remains tentative.

The study was carried out during a single hunting season (2017/2018), which ensured good spatiotemporal comparability between hunting ground types and minimized the impact of climatic and weather-related confounding factors. However, this temporal limitation also prevents the assessment of seasonal and interannual variability in histopathological changes, and the results cannot be directly applied to other years or different climatic conditions without further research.

Sample size and distribution varied between hunting ground types, which may have influenced the statistical power of the analyses. Limited statistical power was especially apparent for rarely occurring histopathological lesions; therefore, findings related to low-prevalence changes should be interpreted with appropriate caution.

Histopathological changes in this study were recorded qualitatively without detailed grading of lesion severity. Although assessments followed a standardized approach, the absence of a semi-quantitative or quantitative scoring system may limit finer differentiation of lesion intensity and full reproducibility in future research.

Despite these limitations, this study offers a useful baseline for future research on histopathological patterns in *L. europaeus* across gradients of human-altered habitats. Future work should combine histopathological data with direct contaminant testing, screening for infectious agents, spatial (geospatial) pollution information, and, if suitable, molecular or biological markers. An integrated approach like this would allow for a more complete understanding of how habitat type, ecological stressors, and the health of *L. europaeus* populations are connected.

## CONCLUSION

The current study showed that specific habitat-related patterns of pathomorphological changes can be observed in the European brown hare (*Lepus europaeus* Pall.) from hunting grounds with different land-use types. Hares from agricultural hunting areas more often displayed dystrophic liver changes and reactive splenic alterations, including splenomegaly and lymphoid hyperplasia. Conversely, hares from periurban or industrial hunting areas had a higher occurrence of lung lesions, especially bronchiolitis and emphysema. Renal lesions appeared with similar frequency in both habitat types, suggesting no clear habitat-specific pattern in the kidneys. These results indicate that various organ systems may react differently to environmental factors linked to agricultural and periurban or industrial habitats.

A major strength of this study is its cross-sectional comparative design conducted within the same geographic region and during a single hunting season, which minimized the effects of seasonal and climatic variability and allowed for more reliable comparisons between habitat types. Additionally, the simultaneous histopathological evaluation of multiple internal organs provided a comprehensive overview of the health status of the studied population, enabling the identification of organ-specific patterns rather than isolated findings. The use of routinely harvested animals also ensured that the results accurately reflect real field conditions and the actual population status, thereby enhancing the practical relevance of the findings for wildlife health monitoring and hunting management.

Although the observed differences cannot be directly linked to specific environmental factors, the results suggest that pathomorphological examination of internal organs can be a valuable complementary tool for assessing the health of European brown hare populations in habitats affected by various forms of human activity. Habitat-related patterns of lesions, especially those involving the liver, spleen, and lungs, may serve as indicative markers of the overall ecological stress experienced by populations, even without direct measurements of contaminants or other stressors.

In conclusion, this study provides evidence that systematic monitoring of the European brown hare’s health can help improve understanding of how habitat features relate to population well-being. This approach may aid wildlife management, conservation efforts, and One Health–focused environmental surveillance by allowing early detection of harmful ecological conditions and directing further focused research on potential environmental stressors.

## DATA AVAILABILITY

The data generated during the study are included in the manuscript.

## AUTHORS’ CONTRIBUTIONS

ZP, DB, VL, and DM: Conceptualization, study design, and methodology. NM, SS, and BS: Fieldwork, sample collection, and transport of specimens to the laboratory. DM: Pathological examinations, sample preparation, laboratory processing, organ examination, and primary interpretation of pathological findings. ZP, NM, and SS: Statistical analysis. ZP and NM: Original manuscript drafting. ZP: Supervision and coordination of the overall research process. ZP, DB, VL, DM, and NM: Interpretation of the results and formulation of recommendations. NM, ZP, and DM: Manuscript revision in response to reviewers’ comments. All authors have read and approved the final version of the manuscript.
